# An online cross-sectional survey of community pharmacists to assess information needs for evidence-based self-medication counselling

**DOI:** 10.1007/s11096-023-01624-7

**Published:** 2023-08-02

**Authors:** J. M. Alexa, T. Bertsche

**Affiliations:** 1 Department of Clinical Pharmacy, Institute of Pharmacy, Faculty of Medicine, Leipzig University, Leipzig, Germany; 2 Drug Safety Center, University Hospital Leipzig and Leipzig University, Bruederstr. 32, 04103 Leipzig, Germany

**Keywords:** Community pharmacy services, Evidence-based pharmacy practice, Information sources, Self care, Self medication, Surveys and questionnaires

## Abstract

**Background:**

Community pharmacists play an important role in healthcare. They are frequently visited by patients to receive advice on self-medication products. Little research has been conducted to investigate pharmacists’ information needs for evidence-based self-medication counselling.

**Aim:**

To assess community pharmacists’ information needs in five predefined areas: general and specific individual needs, quality needs, utilisation needs, implication needs, and access needs for evidence based self-medication counselling.

**Method:**

After ethical approval, we conducted an exploratory, semi-quantitative, cross-sectional online survey. Members of three different chambers of pharmacists in Germany were invited to participate anonymously in the survey. They gave informed consent and received no incentive for their participation.

Quantitative outcome: Frequency of relevance / importance of items within predefined information needs areas, except for access needs. Qualitative outcome: Open-text responses concerning all information needs.

**Results:**

We analysed data from a total of 823 participants who completed the survey. General and specific information such as dosage (74.2% [611/823]) and when to refer to a physician (64.6% [532/823]) as well as an over-the-counter product’s effectiveness according to medical guidelines (71.4% [588/823]) were rated as very important. Participants reported to prefer digital information sources (50.5% [416/823] strongly agreed), especially in the form of an easily accessible database (61.6% [507/823] strongly agreed) that contains regularly updated, manufacturer-independent, critically appraised, concise information.

**Conclusion:**

Community pharmacists expressed distinct information needs for evidence-based self-medication counselling. Further information services on essential evidence-based pharmacy knowledge may be necessary to support implementation.

**Supplementary Information:**

The online version contains supplementary material available at 10.1007/s11096-023-01624-7.

## Impact statements

- The results of our study help to understand what information needs pharmacists have in order to enable evidence-based self-medication counselling.
- The data shed light onto pharmacist-specific preferences, needs and potential knowledge gaps that can serve as a guidance for future information services.
- These findings may help to comprehend how the evidence-to-practice gap can be reduced.

## Introduction

Community pharmacies play an important role in healthcare [1–3]. They are frequently visited by patients to receive well-grounded advice related to self-medication [4, 5]. In various countries over-the-counter products can however also be purchased in different establishments, including grocery stores. Therefore, self-medication counselling represents a key competency of pharmacists [6]. Ideally, evidence-based counselling includes the patient’s preference, the pharmacist’s practical experience and the best available external evidence as is defined by the principles of evidence-based pharmacy (EbPharm). If these criteria are met, pharmacists can achieve best patient outcomes with a positive impact on the entire healthcare system [7, 8].

Previously published studies have shown, however, that external evidence in particular is only sparsely integrated into everyday counselling [9–18]. Barriers such as a lack of time and missing information resources were identified as the main reason causing the evidence-to-practice gap [19–24]. So far, very few measures have been introduced to bridge this gap. Examples comprise the creation of a pharmacist-targeted, evidence-based newsletter and a corresponding database known as the EVInews project [25] as well as educational training interventions [26–32]. It is widely agreed that the implementation of EbPharm in everyday practice can only succeed with practical support [33]. Yet, to our knowledge no previous studies have investigated what information needs pharmacists have in this context. Understanding pharmacists’ information needs is crucial to navigate further steps towards the successful implementation of EbPharm.

The term ‘information needs’ has been used interchangeably, but mainly to describe the types of information health professionals need for their daily work [34–37]. ‘Information’ has been used to refer to the necessary input that removes uncertainties in a decision-making process [37]. Recent investigations [35] have examined subordinate areas of health professionals’ information needs for evidence-based practice such as individual needs, quality needs and access needs.

Our investigation into this gap in the research strived to contribute to a better understanding of community pharmacists’ information needs and how external evidence can be better integrated for evidence-based counselling in community pharmacies. The findings aimed at providing guidance on tailoring information sources and interventions according to pharmacists’ needs.

### Aim

This study aimed to assess community pharmacists’ information needs in five predefined areas: (*1.a) general individual needs, (1.b) specific individual needs, (2) quality needs, (3) utilisation needs, (4) implication needs* and *(5) access needs* for evidence-based self-medication counselling.

### Ethics approval

Ethical approval for this study was granted by the Ethics Committee of the Medical Faculty of Leipzig University in alignment with the Declaration of Helsinki. The approval reference number 423/22-ek was issued on December 6, 2022.

## Method

### Study design

This study was conducted in the form of an exploratory, semi-quantitative, cross-sectional, online survey between December 1, 2022, and January 22, 2023.

### Study sample, setting, and recruitment

**Fig. 1:**
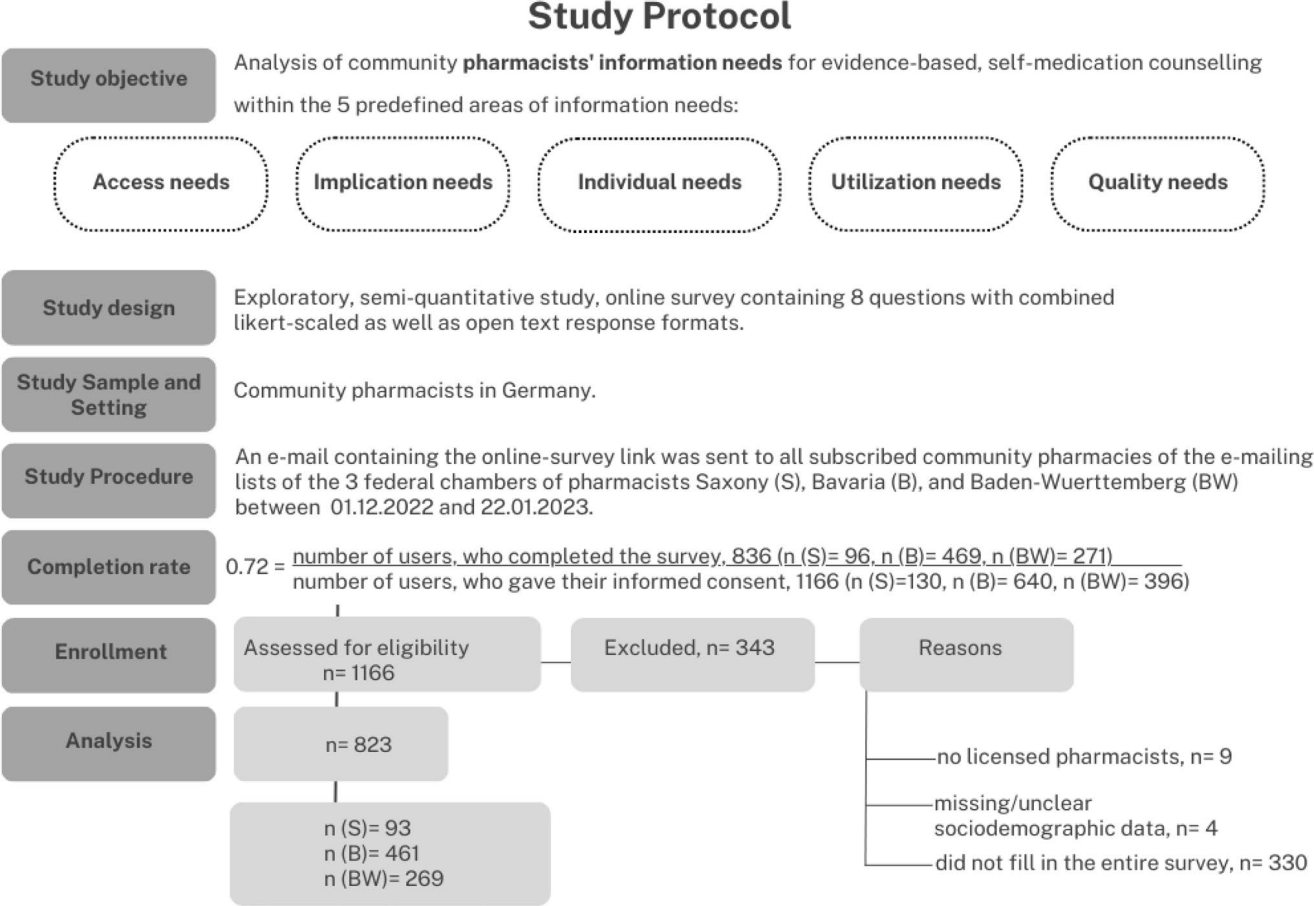
Study protocol including completion rate and participant flow chart, modified based on CROSS, STROBE and CHERRIES [39–41]

The convenience study sample consisted of community pharmacists from three different federal chambers of pharmacists (Saxony, Bavaria, Baden-Wuerttemberg). These chambers were chosen because of their greatly varying pharmacy density, postgraduate education programmes and location in the east, south, and south-west of Germany. The participants were invited via e-mail through each chamber of pharmacists to fill in the web-based survey. An e-mail containing the survey link was sent to all community pharmacies subscribed to each federal chamber of pharmacists’ e-mailing list. The participation was voluntary, anonymous, and no incentives were given. The study protocol and participant flow chart is illustrated in Fig. 1.

SoSci Survey (Version 3.1.06) [38] was used to perform the survey, automatic data collection, and completion assessment. The survey link was accessible at www.soscisurvey.de without any restrictions. Information about the study’s purpose, investigator, estimated length, and data handling was displayed on the first page, before participants gave their written informed consent. Data processing and handling met the European data protection law standards. Since the data collection was anonymous and no cookies were used, multiple data entries were beyond our control. Participants were able to review and alter their responses by using a back-button. A reminder inquiry was sent to every chamber of pharmacists in January 2023.

### Inclusion criteria

Pharmacists had to be licensed pharmacists who had completed the entire survey for their data to be included.

### Outcome parameters

The results of this study are structured into three parts (*Part A, B and C*). These are not corresponding to the original survey structure because the sociodemographic questions were placed at the end of the survey.

Sociodemographic data (*Part A.1*), Correlation analysis (*Part A.2*).

*Quantitative outcome*: Frequency of relevance / importance of items within all information needs areas, except for access needs (*Part B*).

*Qualitative outcome*: Identification of items based on open-text responses concerning access needs and additional items concerning all other areas of information needs (*Part C)*.

### Survey structure

The survey spread over 12 screen pages and was designed to semi-quantitatively analyse pharmacists’ information needs. It was subdivided into three sections, which were as follows:

- Section 1: Introduction containing general information about the study, informed consent, instructions, and a figure presenting the survey’s purpose.
- Section 2: Information needs in five predefined areas for evidence-based over-the-counter (OTC) counselling (eight questions). The five categories of information needs included *(1.a) general individual needs* (one question)*, (1.b) specific individual needs* (one question), *(2) quality needs* (one question), *(3) utilisation needs* (two questions), *(4) implication needs* (two questions), and *(5) access needs* (one question), (*Part B and C*)*,*
- Section 3: Sociodemographic data (ten questions*, Part A.1).*
- Section 2 contained one open-ended question addressing access needs and seven closed questions for all other information needs areas. Participants were invited to quantitatively determine the relevance or importance of items given within the closed-ended questions through 4-point Likert scales and designated text fields. The translated questions can be found within the online supplementary material (S1).

### Survey development and pretesting

The chosen areas of information needs were based on previous findings regarding other health professionals’ information needs for evidence-based practice [34, 35]. We then developed questions that specifically addressed pharmacists and their information needs in the community pharmacy. The online survey was pretested using SoSci Survey in a two-step process with 17 pharmacists. Initially, the think-aloud method was used for 12 pretests to obtain in-depth information about comprehension, feasibility, layout preferences, and how non-response errors could be prevented. After feedback-based modifications, the standard observation method was employed for the remaining pretests. A total of nine pre-testers belonged to the target group of community pharmacists who were involved in daily self-medication counselling. No pre-tester was involved in survey design, conduct, or data analysis. Data generated by the pretests were not included in the final data analysis.

### Data analysis

All analyses were performed using Microsoft Office Excel (version 2016, Microsoft Corporation, Redmond, Washington, U.S.A.) and IBM® SPSS® Statistics version 29.0 (IBM Corporation, Armonk, New York, U.S.A.). The quantitative data analysis included descriptive statistics and a Spearman’s rank order correlation to investigate the relationship between sociodemographic traits and familiarity with EbPharm-related topics (*Part A.2*). Furthermore, the comparability of the participants’ sociodemographic characteristics between the three chambers of pharmacists was tested using a two-sided Kruskall-Wallis test and a Chi-square test. The threshold for statistical significance was set at *p* < 0.05.

Data generated by this survey comprised qualitative data and ordinal or nominal scaled data. Text responses to open-ended questions were thematically and manually clustered in a two-step process into variables based on a deductive, hierarchical approach. The variables’ frequencies were determined afterwards. In uncertain cases, the respective variable was discussed between the two study authors and clustered based on consensus or excluded from the analysis.

We estimated that 100 participants would provide a representative basis for our data analysis in each chamber of pharmacists. Data from participants who provided non-analysable data or did not complete the survey were excluded.

## Results

### Study protocol and response rate

The study protocol and completion ratio is presented in Fig. 1.

### (Part A.1) Participants’ characteristics

The participants’ characteristics are provided in Table 1. A total of 823 pharmacists fulfilled the inclusion criteria.

**Table 1:** Sociodemographic data of the study sample

	Characteristics	Study sample (n = 823)
Gender [n (*%*)]	Female	684 (*83.1)*
	Male	139 (*16.9*)
	Nonbinary	0 *(0.0)*
Age [years]	Median age, IQR	45.0 (*35.0 – 54.0*)
Academic degree [n (*%*)]	State Examination only	747 (*90.8*)
	Additional Diploma (Pharmacy)	24 (*2.9*)
	Additional Doctorate	50 (*6.1*)
	Additional Master of science	1 (*0.1*)
	Additional Diploma (others, e.g. Chemistry)	1 (*0.1*)
Additional qualifications [n (%)]	Participants with additional qualifications	196 *(23.8)*
	Pharmacy specialist in the field of:	
	General pharmacy	88 (10.7)
	Clinical pharmacy	9 (1.1)
	Drug information	6 (0.7)
	Pharmaceutical analytics and technology	2 (0.2)
	Educational training in the area of:	
	Geriatric pharmacy	46 (*5.6)*
	Natural remedy and homeopathy	34 *(4.1)*
	Medication management and medication therapy safety	24 *(2.9)*
	Nutrition counselling	26 *(3.2)*
	Prevention and health promotion	8 *(1.0)*
	Oncological pharmacy	5 *(0.6)*
	Other	31 (3.8)
Work Experience [years] [n (*%*)]	≤ 1	34 (*4.1*)
	2–5	120 (*14.6)*
	6–10	107 (*13.0*)
	≥ 11	562 (*68.3*)
Previous Work Experience in different Pharmaceutical Fields [n (*%*)]	Community pharmacy	823 (*100.0*)
	Hospital pharmacy	62 (*7.5*)
	Teaching and research	41 (*5.0*)
	Pharmaceutical industry	42 (*5.1*)
	Public offices and authorities	3 (*0.4*)
	Health insurance	1 (*0.1*)
	Federal armed forces	1 (*0.1*)
	Other	17 (*2.1*)
Current Job Position in Community Pharmacy [n (*%*)]	Pharmacy owner	151 (*18.4*)
	Branch manager	113 (*13.7*)
	Employee	552 (*67.1*)
	Other	7 (*0.9*)
Currently involved in self-medication counselling [n (*%*)]	Yes	805 (*97.8)*
	No	18 (*2.2*)
Age and educational differences between the chambers of pharmacists	Saxony (S), age: 36 years (IQR: 31.0 – 47.5), Diploma in pharmacy (Dipl.-Pharm.): 15.1% (14/93), Doctoral degree 9.7% (9/93)	
	Bavaria (B), age: 46 (IQR: 36.0 – 55.0), Diploma in pharmacy (Dipl.-Pharm.): 1.1% (5/461), Doctoral degree: 4.6% (21/461)	
	Baden-Wuerttemberg (BW), age: 46 (IQR: 35.0 – 55.0), Diploma in pharmacy (Dipl.-Pharm.): 0.7% (2/269), Doctoral degree: 6.7% (18/269)	

A Kruskall-Wallis test showed statistically significant sociodemographic differences between the participants of the three different chambers of pharmacists concerning age, academic degree (*p* < 0.001 each) and professional experience (*p* = 0.047). We did not identify any statistically significant differences regarding educational training and gender.

### (Part A.2) Correlation analysis

The results of the correlation analysis are shown in Table S3 (online supplementary material S3). Increasing professional experience was associated with a decrease in familiarity with all five EbPharm-related topics (weak, negative correlation with values ranging from r_s_ = − 0.080 to r_s_ = − 0.205 and *p* ≤ 0.022 respectively), the use of external evidence (r_s_ = − 0.122, *p* < 0.001) and the interpretation of study results (r_s_ = − 0.123, *p* < 0.001).

### Quantitative outcome

#### (Part B) Quantitative assessment of pharmacists’ information needs for evidence-based counselling

**Fig. 2:**
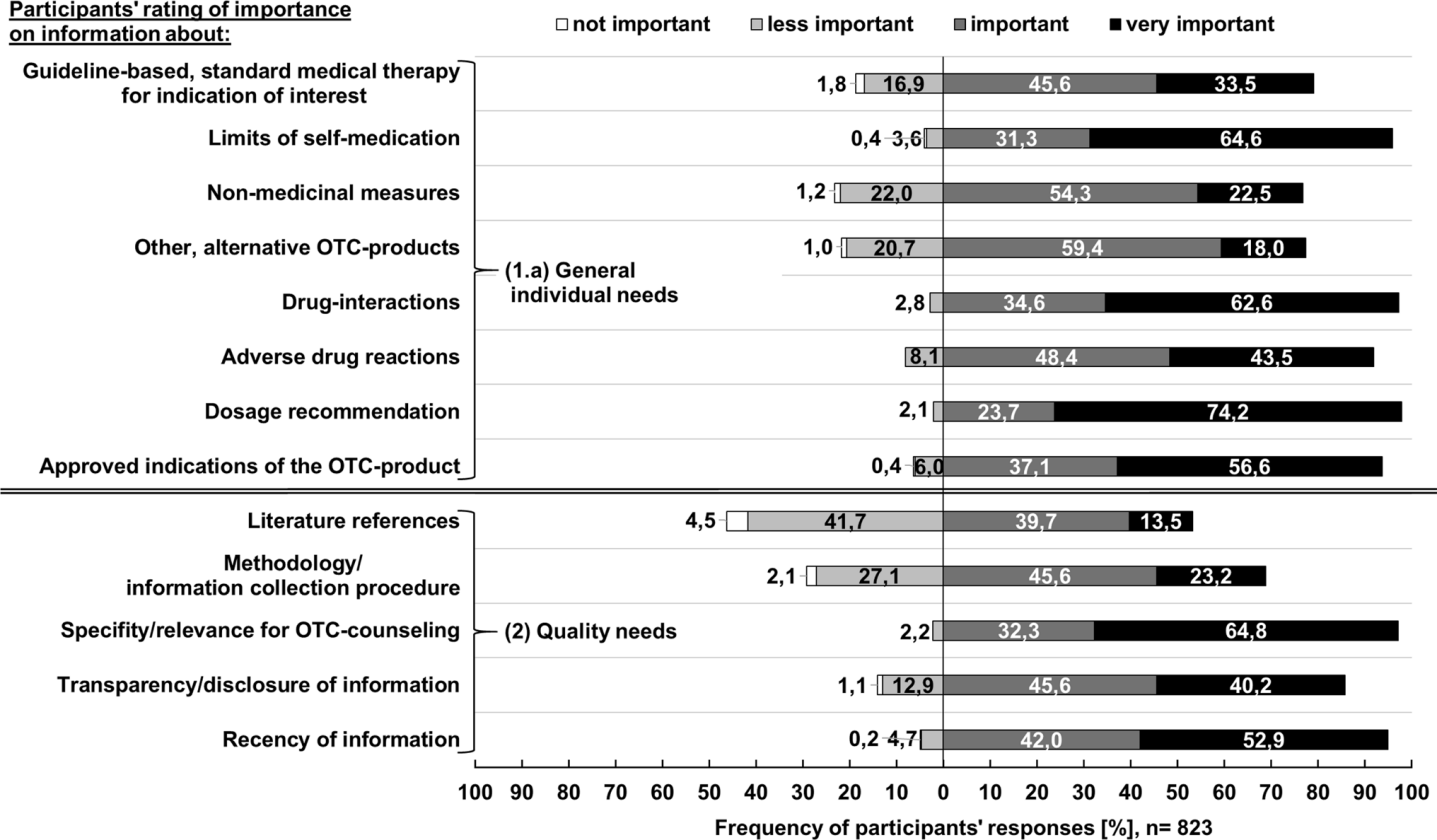
Participants’ rating of importance of general OTC-counselling related information for pharmacists ([1.a] general individual needs) and of quality criteria for OTC-related information sources external evidence ([2] quality needs)

**Fig. 3:**
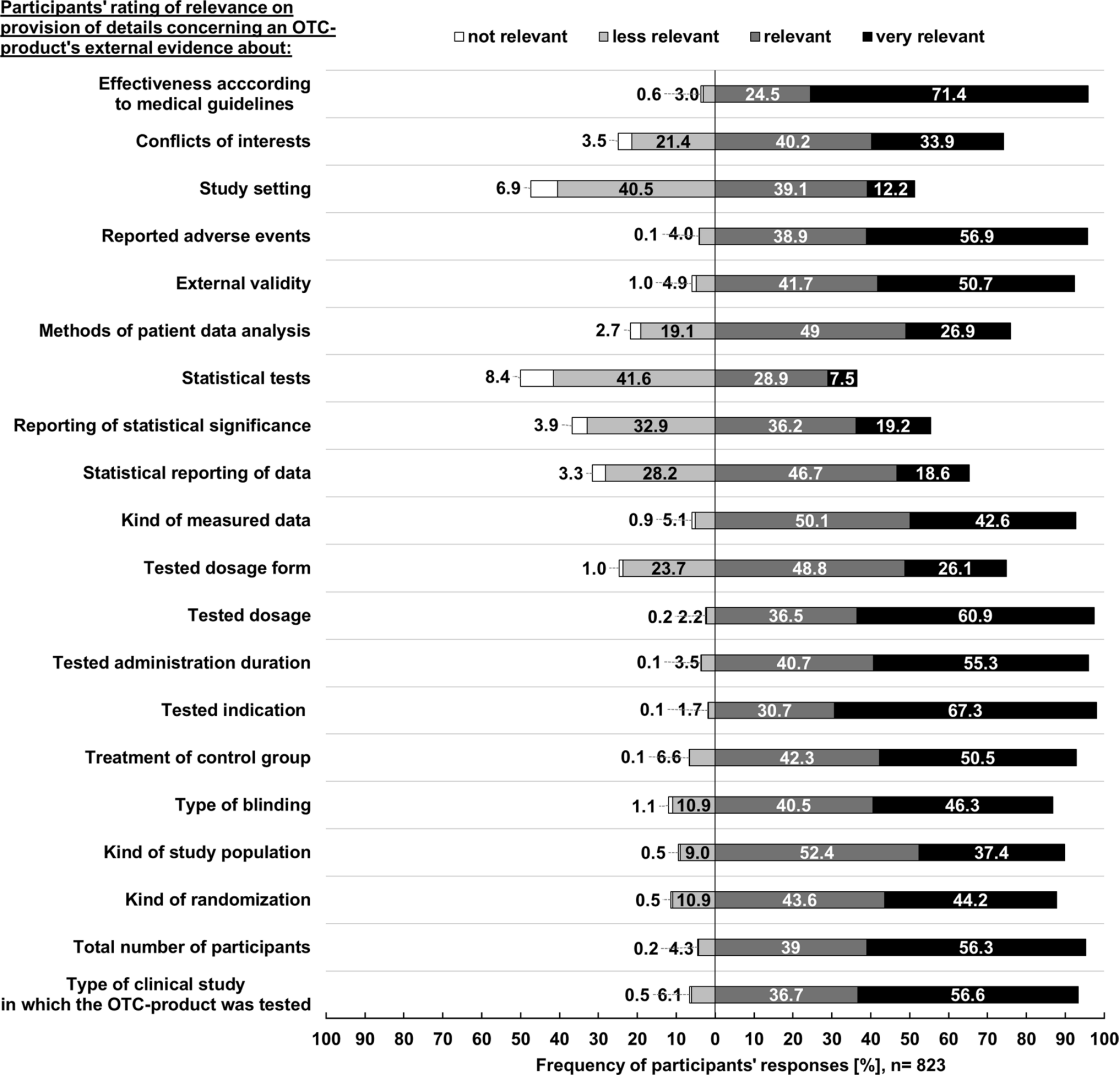
Participants’ rating of relevance on the provision of information regarding OTC-related external evidence ([1.b] specific individual needs)

**Fig. 4:**
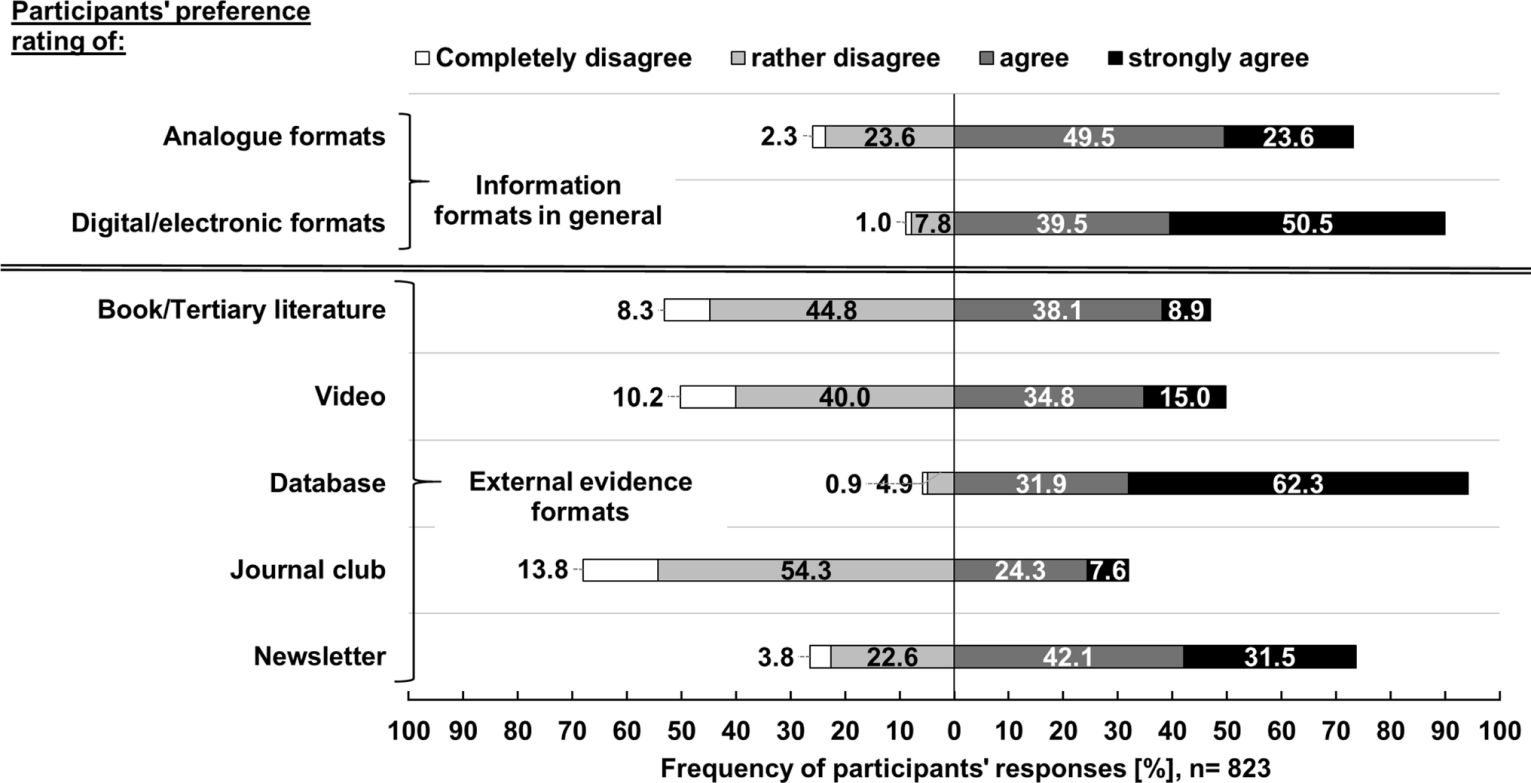
Participants’ rating of agreement on preferred information formats in general and external evidence formats ([3] utilization needs)

**Fig. 5:**
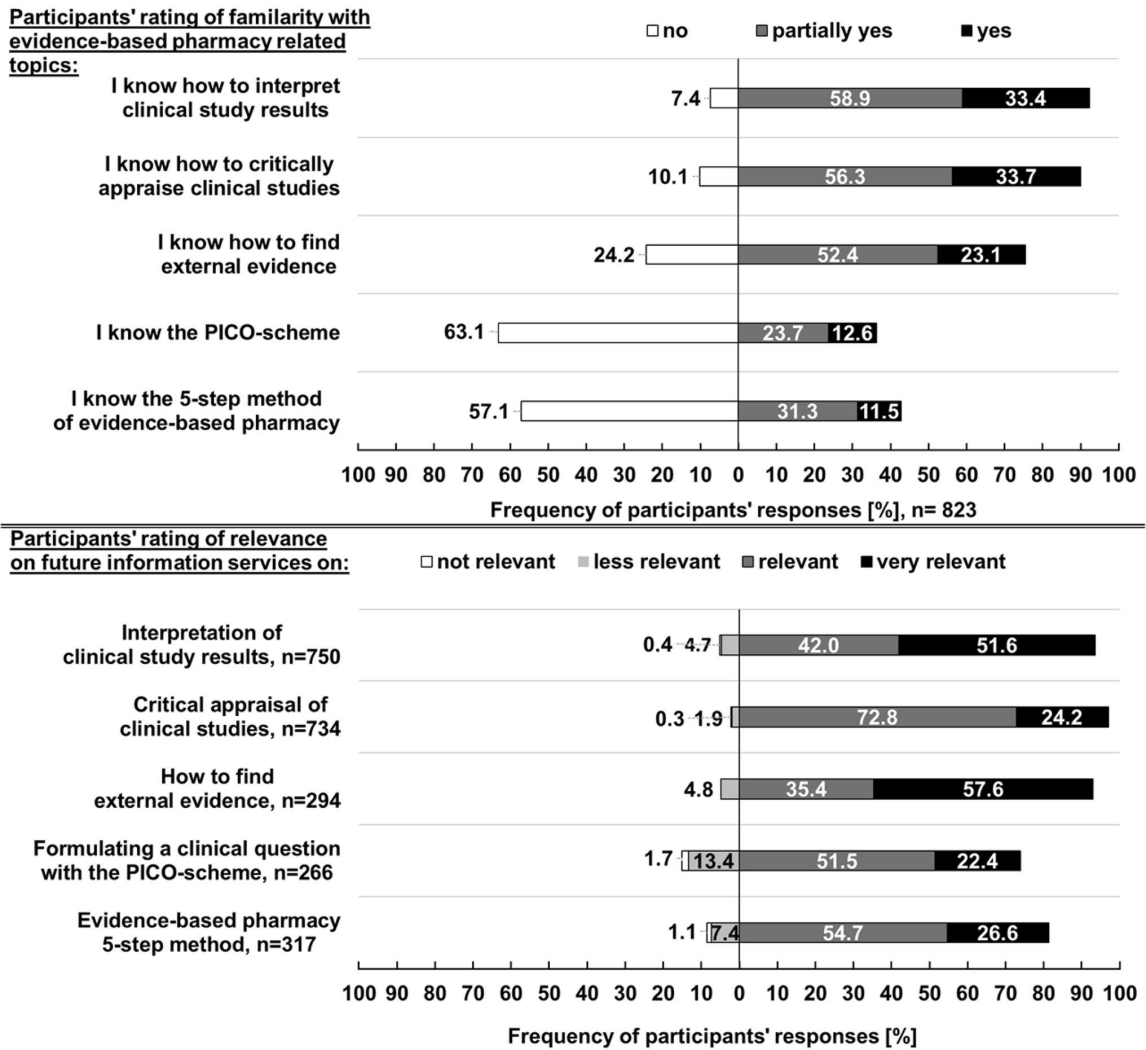
Participants’ rating of familarity EbPharm-related topics and rating of relevance of these for future information services ([4] implication needs). For the rating of relevance, only responses from participants who indicated to be at least partially familiar with the topic were considered

Figures 2, 3, 4 and 5 illustrate the quantitative survey results.

As can be seen from Fig. 2, more than half of all participating pharmacists rated general information about dosage (74.2% [611/823]) and drug interactions (62.6% [515/823]) to be very important for counselling. Many participants indicated that information concerning effectiveness according to medical guidelines (71.4% [588/823]) and tested indications (67.3% [554/823]) were very relevant (Fig. 3).

Opinions on the importance of information source quality criteria varied. More than two-thirds of all participants found relevance for OTC counselling (64.8% [533/823]) and recency (52.9% [435/823]) to be very important. Closer inspection of Fig. 4 shows that 50.5% (416/823) strongly prefer digital/electronic information formats, particularly databases (61.6% [507/823]). Only a small number of participants reported to be not familiar with the interpretation of clinical studies (7.4% [61/823]) and critical appraisal (10.1% [83/823]). Participants who indicated to be at least partially familiar with the five given topics felt that the interpretation of clinical study results (52.3% [392/750]) and how to find external evidence (61.2% [180/294]) were very relevant for future information services.

### Qualitative outcome:

#### (Part C) Cluster analysis of the open-text responses

The results from the cluster analysis of the open-text responses are shown in Table S2 (online supplementary material S2).

- General individual needs: A total of five respondents suggested that practical experience (40.0% [2/5]), usage during pregnancy and while breastfeeding (20.0% [1/5]) as well as abuse and misuse potential (20.0% each [1/5]) were important information for OTC counselling in general.
- Specific individual information needs: Six participants added other information they found relevant regarding an OTC product’s external evidence, which include: conflicts of interests in medical guidelines, time since market authorisation, long-term monitoring data and independence of study results (16.7% each [1/6]).
- Quality needs: A total of five out of 16 participants mentioned details about study characteristics (31.3% [5/16]) as another quality criterion for counselling-related information sources. Nearly 20% (18.8% each [3/16)] also disclosed independence, clarity and conciseness to be important quality traits.
- Utilisation needs: Out of all 56 open-text responses, webinars (17.9% [10/56]), summaries (10.7% [6/56]) as well as free and easy-to-use databases (8.9% [5/56]) emerged as the most frequently mentioned electronic formats. Moreover, 16.1% (9/56) of respondents named podcasts as a potential audio information format, and a smaller proportion mentioned analogue formats such as ring binders (5.4% [3/56]).
- Implication needs: The search for information is a theme that emerged from the three given open-text responses. Participants mentioned the search for independent information sources in general, for independent studies and for information about the EbPharm methodology/concept (33.3% each [1/3]).
- Access needs: Less than half of all participants (43.0% [354/823]) provided answers for the open-ended question about how to improve the access to OTC-related external evidence for pharmacists. The establishment of a database was the most frequent response (26.8% [95/354]). The second most frequently given response concerned the linking of evidence-based information/databases with the pharmacy software (13.6% [48/354]), followed by the provision of short summaries/overviews (12.4% [44/354]). The majority of participants’ responses mentioned information formats or measures that require an active search (295/479). The denominator 479 represents the frequency of how often an identified variable was mentioned.

## Discussion

### Statement of key findings

Community pharmacists play an important role as health promoters in healthcare, particularly for self-medication management. Little attention has been paid to their information needs for evidence-based self-medication counselling. Previous research mainly focused on investigating barriers, educational interventions and the status quo of counselling practice [9, 12, 13, 19, 20, 42]. This study set out with the aim of gaining an insight into their information needs in five predefined areas to help implement EbPharm into practice. The majority of participants indicated that the establishment of an information tool, preferably a database, containing regularly updated, independent, critically appraised, concise and relevant information would be useful to meet their information needs. Information such as dosage, interactions, and effectiveness of an OTC product according to medical guidelines were rated as very important. Digital formats that allow an active search for information were preferred. Further educational training on EbPharm basics may be necessary to enable a correct usage of the implementation of such sources in EbPharm.

### Strengths and weaknesses

To our knowledge, this is the first study that investigated the information needs of community pharmacists for evidence-based self-medication counselling. As described by previous studies [34, 35, 43], health professionals’ information needs vary greatly given the different scope of practice and professional demands they face. We therefore questioned a large sample of community pharmacists (n = 823) directly by using a semi-quantitative approach. Participants from different chambers of pharmacists and therefore regions in Germany were included, of whom 97.8% [805/823] reported to be involved in self-medication counselling. Information needs are dependent on working conditions, not location. The findings of our study may therefore be relevant for policy makers and the pharmacist workforce.

We examined five areas of pharmacists’ information needs. Other areas of information needs remain to be explored. Furthermore, it was impossible to include all potentially relevant items in the Likert-scaled responses. However, participants were given the opportunity to add new items as open-text responses. The study sample moreover included participants from three out of 17 different chambers of pharmacists. Additionally, it was impossible to identify unique site visitors given that the survey was anonymous.

### Interpretation

#### Sociodemographic differences

A closer inspection of the significant differences between the three chambers of pharmacists revealed that these discrepancies were attributed to a lower median of age, more participants holding a pharmacy diploma and fewer doctoral degrees in the chamber of Saxony in comparison to the others. Diploma titles were previously only available in the former German Democratic Republic (GDR) and mainly universities located in the east of Germany offer such degrees to this day.

#### Information needs and correlation analysis

The results concerning the information needs seem plausible, since a well-grounded product recommendation requires a thorough assessment of its appropriateness, benefits and potential harm for the patient. OTC products such as medicines or supplements are authorised on the market in various ways. The manufacturers are not always obliged to provide sufficient or even any clinical data at all, which complicates counselling-related information retrieval. According to open-text responses, participants also expressed the desire to use information sources that already contain manufacturer-independent, critically appraised information. This should be considered when customising information sources for pharmacists for evidence-based self-medication counselling.

Interestingly, the majority of participants reported to prefer digital formats in general, especially a free and easy-to-access database, when asked about their utilisation needs. They would rather use formats that enable an active search instead of a passive information supply. This observation may be attributed to the fact that when confronted with OTC-related inquiries, pharmacists are expected to access the required information quickly.

Surprisingly, when asked to evaluate the importance of quality criteria of external evidence, most participants rated relevance for counselling as very important, but not disclosure of literature references. Moreover, two thirds of all participants indicated to be at least partially familiar with, for instance, critical appraisal of clinical studies. Yet, more than half of all participants were not familiar with basic subjects such as the PICO-format. This appears to be contradictory, but may be due to a gap of knowledge. EbPharm has only been introduced in recent years and a great proportion of participants had more than ten years of professional experience. The correlation analysis also showed a weak negative relationship between the familiarity with fundamental EbPharm-related topics and professional experience as well as the participants’ academic degree. Participants with postgraduate education perhaps scrutinised their skillset more realistically. As already suggested [15, 17, 19], measures to increase knowledge regarding the EbPharm concept and especially external evidence should be intensified in the upcoming years.

### Further research

Areas of future research should investigate additional areas of information needs to gain a deeper insight. After the creation of an information tool that is tailored to pharmacists’ information needs, it should be investigated how the correct usage of external evidence made available by such a tool can be facilitated to reduce the evidence-to-practice gap.

## Conclusion

This study revealed that pharmacists have very specific information needs. They indicated to prefer digital or electronic information source formats, especially in form of an easily accessible database that contains relevant information for evidence-based counselling. Information should fulfill distinct criteria such as being manufacturer-independent, critically appraised and concise. Efforts to provide essential EbPharm-related knowledge should be intensified to ascertain the correct implementation of evidence-based self-medication counselling.

### Supplementary Information

Below is the link to the electronic supplementary material.

Supplementary file1 (DOCX 72 KB)

## References

[1] Pizetta B, Raggi LG, Rocha KSS (2021). Does drug dispensing improve the health outcomes of patients attending community pharmacies? A systematic review. BMC Health Serv Res.

[2] Rutter P (2015). Role of community pharmacists in patients’ self-care and self-medication. Integr Pharm Res Pract..

[3] ABDA—Federal Union of German Associations of Pharmacists. German Pharmacies: Figures Data Facts 2022 [English]; 2022. https://www.abda.de/en/. Accessed 27 May 2023.

[4] Nunes FG, Anderson JE, Martins LM (2015). Patient reactions to community pharmacies’ roles: evidence from the Portuguese market. Health Expect.

[5] Hayashi M, Masuda S, Kimura H (2015). Key information providers, channels, and characteristics of Japanese consumers’ informed choices of over-the-counter medications. Springerplus.

[6] van Eikenhorst L, Salema NE, Anderson C (2017). A systematic review in select countries of the role of the pharmacist in consultations and sales of non-prescription medicines in community pharmacy. Res Social Adm Pharm.

[7] Watson MC, Ferguson J, Barton GR (2015). A cohort study of influences, health outcomes and costs of patients’ health-seeking behaviour for minor ailments from primary and emergency care settings. BMJ Open.

[8] Sackett DL, Rosenberg WM, Gray JA (1996). Evidence based medicine: what it is and what it isn’t. BMJ.

[9] Aloudah N, Alhumsi A, Alobeid N (2020). Factors impeding the supply of over-the-counter medications according to evidence-based practice: a mixed-methods study. PLoS ONE.

[10] Halila GC, Junior EH, Otuki MF (2015). The practice of OTC counseling by community pharmacists in Parana. Brazil Pharm Pract (Granada)..

[11] Moritz K, Seiberth JM, Schiek S (2019). The impact of evidence from clinical trials on counselling for over-the-counter drugs: a national survey of pharmaceutical staff in German pharmacies. J Clin Pharm Ther.

[12] Abu Farha R, Alefishat E, Suyagh M (2014). Evidence-based medicine use in pharmacy practice: a cross-sectional survey. J Eval Clin Pract.

[13] Alefishat E, Jarab AS, Muflih S (2022). Community pharmacists’ attitudes toward practice-based research and their perceived utilization of scientific evidence. PLoS ONE.

[14] Hanna LA, Hughes CM (2010). ‘First, do no harm’: factors that influence pharmacists making decisions about over-the-counter medication: a qualitative study in Northern Ireland. Drug Saf.

[15] Johnson H (2013). Selling evidence over the counter: do community pharmacists engage with evidence-based medicine?. Med Writ..

[16] McKee P, Hughes C, Hanna LA (2015). Views of pharmacy graduates and pharmacist tutors on evidence-based practice in relation to over-the-counter consultations: a qualitative study. J Eval Clin Pract.

[17] Paravattil B, El Sakrmy N, Shaar S (2018). Assessing the evidence based medicine educational needs of community pharmacy preceptors within an experiential program in Qatar. Curr Pharm Teach Learn.

[18] Rutter P, Wadesango E (2014). Does evidence drive pharmacist over-the-counter product recommendations?. J Eval Clin Pract.

[19] Al-Jamei S, Abu Farha R, Zawiah M (2019). Perceptions, knowledge, and perceived barriers of Yemeni pharmacists and pharmacy technicians towards evidence-based practice. J Eval Clin Pract.

[20] Burkiewicz JS, Zgarrick DP (2005). Evidence-based practice by pharmacists: utilization and barriers. Ann Pharmacother.

[21] Lafuente-Lafuente C, Leitao C, Kilani I (2019). Knowledge and use of evidence-based medicine in daily practice by health professionals: a cross-sectional survey. BMJ Open.

[22] Ozaki AF, Nakagawa S, Jackevicius CA (2019). Cross-cultural comparison of pharmacy students’ attitudes, knowledge, practice, and barriers regarding evidence-based medicine. Am J Pharm Educ.

[23] Tan SY, Hatah E (2017). Knowledge, attitudes, practices, and barriers related to research utilization: a survey among pharmacists in Malaysia. Int J Clin Pharm.

[24] der Lelie-van Zande R, Koster ES, Teichert M (2023). Barriers and facilitators for providing self-care advice in community pharmacies: a qualitative study. Int J Clin Pharm.

[25] Moritz K, Seiberth JM, Schiek S (2021). Evidence-based self-medication: development and evaluation of a professional newsletter concept for community pharmacies. Int J Clin Pharm.

[26] Laven A, Schäfer J, Läer S. PHARMAGRIPS: Pharmazeutische Beratung in der Selbstmedikation des grippalen Infekts. Eine randomisierte kontrollierte Studie (RCT). Med Monatsschr Pharm 2014; 37:209–20. 25051811

[27] Aoshima S, Kuwabara H, Yamamoto M (2017). Behavioral change of pharmacists by online evidence-based medicine-style education programs. J Gen Fam Med..

[28] Hanna LA, Hughes C (2012). The influence of evidence-based medicine training on decision-making in relation to over-the-counter medicines: a qualitative study. Int J Pharm Pract.

[29] Margolis A, Shah S, Kraus C (2020). Longitudinal assessment of pharmacy students’ confidence and skill in providing evidence-based answers to clinical questions. Am J Pharm Educ.

[30] Neill KK, Johnson JT (2012). An advanced pharmacy practice experience in application of evidence-based policy. Am J Pharm Educ.

[31] Paravattil B, Shabana S, Rainkie D (2019). Evaluating knowledge, skills, and practice change after an accredited evidence-based medicine course for community pharmacy preceptors. Curr Pharm Teach Learn.

[32] Watson MC, Bond CM, Grimshaw JM (2002). Educational strategies to promote evidence-based community pharmacy practice: a cluster randomized controlled trial (RCT). Fam Pract.

[33] ABDA—Federal Union of German Associations of Pharmacists. Pharmacy 2030: Perspectives on provision of pharmacy services in Germany: English [perspective paper]; 2015. Available from: URL: https://www.abda.de/en/. Accessed on 27 Mar 2023.

[34] Barr-Walker J (2017). Evidence-based information needs of public health workers: a systematized review. J Med Libr Assoc.

[35] van der Keylen P, Tomandl J, Wollmann K (2020). The online health information needs of family physicians: systematic review of qualitative and quantitative studies. J Med Internet Res.

[36] Timmins F (2006). Exploring the concept of ‘information need’. Int J Nurs Pract.

[37] Revere D, Turner AM, Madhavan A (2007). Understanding the information needs of public health practitioners: a literature review to inform design of an interactive digital knowledge management system. J Biomed Inform.

[38] Leiner DJ. SoSci Survey: Version 3.1.06; 2019. Available from: URL: https://www.soscisurvey.de. Accessed on 23 Mar 2023.

[39] Sharma A, Minh Duc NT, Luu Lam Thang T (2021). A consensus-based checklist for reporting of survey studies (CROSS). J Gen Intern Med.

[40] von Elm E, Altman DG, Egger M (2008). The strengthening the reporting of observational studies in epidemiology (STROBE) statement: guidelines for reporting observational studies. J Clin Epidemiol.

[41] Eysenbach G (2004). Improving the quality of Web surveys: the checklist for reporting results of internet e-surveys (CHERRIES). J Med Intern Res..

[42] Moritz K, Seiberth JM, Herrmann NS (2021). Are evidence-based criteria addressed during counseling on over-the-counter products? An observational study in community pharmacies. Patient Educ Couns.

[43] Weng YH, Kuo KN, Yang CY (2013). Implementation of evidence-based practice across medical, nursing, pharmacological and allied healthcare professionals: a questionnaire survey in nationwide hospital settings. Implement Sci.

