# Supplementary material for: An online cross-sectional survey of community pharmacists to assess information needs for evidence-based self-medication counselling
Source: Int J Clin Pharm. 2023 Aug 2;45(6):1452–63. doi: 10.1007/s11096-023-01624-7 (PMC10682211; doi:10.1007/s11096-023-01624-7)
Supplement: Supplementary file 1 — Supplementary file1 (DOCX 72 KB) [file 11096_2023_1624_MOESM1_ESM.docx]

**Supplementary material**

**Supplementary material S1** Survey questions

**An online cross-sectional survey investigating community pharmacists’ information needs for evidence-based OTC-counselling**

International Journal of Clinical Pharmacy

Alexa JM, Bertsche T

Dept. of Clinical Pharmacy, Institute of Pharmacy, Faculty of Medicine, Leipzig University and Drug Safety Center, University Hospital Leipzig and Leipzig University, Leipzig, Germany; email to [thilo.bertsche@uni-leipzig.de](mailto:thilo.bertsche@uni-leipzig.de)

Translated questions based on the original survey in German.

**Eight Questions about information needs**

**1**. **From your point of view, which quality criteria are important for evidence-based information sources (external evidence) for pharmacists for evidence-based self-medication counselling?**

external evidence = scientific data

| **Recency** (e.g. publication date, inclusion of recent studies). | not important | less important | important | very important | I don’t know |
| --- | --- | --- | --- | --- | --- |
| **Transparency** (e.g. disclosure of authors, author’s conflicts of interest) | not important | less important | important | very important | I don’t know |
| **Specificity** (e.g. indication of relevant information for the consultation) | not important | less important | important | very important | I don’t know |
| **Methodology** (e.g. systematic approach of the authors when creating the information source) | not important | less important | important | very important | I don’t know |
| **Disclosure of literature sources**  (e.g. within the list of references) | not important | less important | important | very important | I don’t know |
| **Other** | (Open-text response), hint: Multiple entries are possible | | | | |

**2**. **Which of the following information about the external evidence of an OTC product is relevant for pharmacists from your point of view?**

OTC product = over-the-counter (non-prescription) product

external evidence = scientific data

| **Type(s) of clinical trial(s),** in which the OTC product was tested.  (e.g. randomized controlled trial, case series) | not relevant | less relevant | relevant | very relevant | I don’t know |
| --- | --- | --- | --- | --- | --- |
| **Total number of study participants**  (e.g. 100 patients) | not relevant | less relevant | relevant | very relevant | I don’t know |
| **Randomization of participants**  (e.g. were the study participants randomly assigned to the respective study groups). | not relevant | less relevant | relevant | very relevant | I don’t know |
| **Tested Study populations** (e.g. gender, age groups) in (a) clinical trial(s) | not relevant | less relevant | relevant | very relevant | I don’t know |
| **Blinding of participants**  (e.g. were investigators, study participants blinded). | not relevant | less relevant | relevant | very relevant | I don’t know |
| **Medication/**treatment of the control group  (e.g. placebo) | not relevant | less relevant | relevant | very relevant | I don’t know |
| **Tested indication** of the OTC product in (a) clinical trial(s) (e.g. Ginkgo biloba for Alzheimer's dementia) | not relevant | less relevant | relevant | very relevant | I don’t know |
| **Tested administration duration** of the OTC product in (a) clinical trial(s) (e.g. 2 weeks) | not relevant | less relevant | relevant | very relevant | I don’t know |
| **Tested dosages** of an OTC product in (a) clinical trial(s).(e.g. 2 times per day) | not relevant | less relevant | relevant | very relevant | I don’t know |
| **Tested formulations/dosage forms** in (a) clinical trial(s) (e.g. as tablets) | not relevant | less relevant | relevant | very relevant | I don’t know |
| **Type of measured data (endpoints)** in clinical trial(s). (e.g. blood pressure, satisfaction) | not relevant | less relevant | relevant | very relevant | I don’t know |
| **Statistical reporting of measured data** as statistical quantities (endpoints) in clinical study(s) (e.g. relative risk) | not relevant | less relevant | relevant | very relevant | I don’t know |
| **Statistical significance**  (e.g. reporting of p-values) | not relevant | less relevant | relevant | very relevant | I don’t know |
| **Statistical tests** used in (a) clinical trial(s).  (e.g. one-sided or two-sided hypothesis tests) | not relevant | less relevant | relevant | very relevant | I don’t know |
| **Methods of patient data analysis** in (a) clinical trial(s) (e.g. inclusion of all study participants or inclusion of those, who completed the study). | not relevant | less relevant | relevant | very relevant | I don’t know |
| **Transferability of the study results** onto the client‘s inquiry (external validity) | not relevant | less relevant | relevant | very relevant | I don’t know |
| **Adverse events** that occurred in (a) clinical trial(s) (ADE) (e.g. skin rash) | not relevant | less relevant | relevant | very relevant | I don’t know |
| **Study setting** of the clinical trial(s) (e.g. hospital, medical office) | not relevant | less relevant | relevant | very relevant | I don’t know |
| **Study authors‘ disclosure on conflicts of interests** (e.g. funding through manufacturer) | not relevant | less relevant | relevant | very relevant | I don’t know |
| **Estimated effectiveness of the OTC product according to medical guidelines** | not relevant | less relevant | relevant | very relevant | I don’t know |
| **Other** | (Open-text response), hint: Multiple entries are possible | | | | |

**3**. **How important do you find the following general information for the consultation, when informing about an OTC-product for a customer inquiry?**

OTC product = over-the-counter (non-prescription) product

| **Approved indications** of the product | not important | less important | important | very important | I don’t know |
| --- | --- | --- | --- | --- | --- |
| **Dosage recommendation** | not important | less important | important | very important | I don’t know |
| **Adverse drug reactions** | not important | less important | important | very important | I don’t know |
| **Drug-interactions** | not important | less important | important | very important | I don’t know |
| **Other, alternative OTC-products** | not important | less important | important | very important | I don’t know |
| Additional, **non-medicinal measures** | not important | less important | important | very important | I don’t know |
| **Limits of self-medication medication** | not important | less important | important | very important | I don’t know |
| **Guideline-based, standard medical therapy for indication of interest** | not important | less important | important | very important | I don’t know |
| **Other** | (Open-text response), hint: Multiple entries are possible | | | | |

**4**. **Which of the following formats for external evidence of OTC products for self-medication counselling do you prefer in general?**

external evidence = scientific data

OTC product = over-the-counter (non-prescription) product

| **Digital/electronic formats** (e.g. databases) | completely disagree | rather disagree | agree | strongly agree | I don’t know |
| --- | --- | --- | --- | --- | --- |
| **Analog formats** (e.g. books, specialized journals) | completely disagree | rather disagree | agree | strongly agree | I don’t know |

**5**. **Which of the following formats of external evidence for OTC-product-counselling would you like (to use) for your everyday professional practice in the future?**

external evidence = scientific data

| **Newsletter** (regularly published information circular) | completely disagree | rather disagree | agree | strongly agree | I don’t know |
| --- | --- | --- | --- | --- | --- |
| **Database** (Electronic management system of data files) | completely disagree | rather disagree | agree | strongly agree | I don’t know |
| **Journal Club** (meetings, in which scientific papers are presented and critically appraised) | completely disagree | rather disagree | agree | strongly agree | I don’t know |
| **Video** (electronically recorded movie) | completely disagree | rather disagree | agree | strongly agree | I don’t know |
| **Book/Tertiary literature** (collection of text sections) | completely disagree | rather disagree | agree | strongly agree | I don’t know |
| **Other** | (Open-text response), hint: You can also name your preferred formats, if not listed. Multiple entries are possible | | | | |

**6**. **Do you know the following subject areas?**

| **I know the EbPharm 5-step method.** | no | partially | yes |
| --- | --- | --- | --- |
| **I know the PICO-scheme.** | no | partially | yes |
| **I know how to find external evidence.** | no | partially | yes |
| **I know how to critically appraise clinical trials.** | no | partially | yes |
| **I know how to interpret study results.** | no | partially | yes |

**7**. **Which of the following subject suggestions for future information services for pharmacists concerning external evidence of OTC products do you find relevant?**

external evidence = scientific data

OTC product = over-the-counter (non-prescription) product

| **General procedure in EbPharm (5-step method)** | not relevant | less relevant | relevant | very relevant | I don’t know |
| --- | --- | --- | --- | --- | --- |
| **How do I pose a precise, clinical question based on the PICO-scheme?** | not relevant | less relevant | relevant | very relevant | I don’t know |
| **Where can I find external evidence within (the framework of) everyday pharmacy practice?** | not relevant | less relevant | relevant | very relevant | I don’t know |
| **How do I critically appraise clinical trials with regards to trustworthiness?** | not relevant | less relevant | relevant | very relevant | I don’t know |
| **How do I interpret study results?** | not relevant | less relevant | relevant | very relevant | I don’t know |
| **Other** | (Open-text response), hint: You can also name your subjects of preference. Multiple entries are possible | | | | |

**8**. **What could be improved from your point of view, to ease the access to external evidence of OTC-products for everyday pharmacy practice?**

external evidence = scientific data

| **Please elaborate** | (Open-text response), hint: Multiple entries are possible. | I don’t know |
| --- | --- | --- |

**9. Do you have any comments or suggestions that you would like to share about the questionnaire or individual questions?**

| **No** | |
| --- | --- |
| **Yes** | (Open-text response), hint: Please explain |

**Sociodemographic questions**

In order to be able to analyze your answers, we need your sociodemographic information. The data collection is anonymous and does not allow any conclusion about your identity. Please answer the following questions by clicking on the corresponding answer.

**10. What is your gender?**

| **female** |  |
| --- | --- |
| **male** |  |
| **divers** |  |

**11. How old are you?**

| **Please enter your age in years.** | (Open-text response) |
| --- | --- |

**12. Which training did you last complete?**

| **Pharmaceutical technical assistant** |  |
| --- | --- |
| **Pharmaceutical engineer** |  |
| **Pharmacist** |  |
| **Other** | (Open-text response), hint: Job title |

**12. Which training did you last complete?**

| **Pharmaceutical technical assistant** |  |
| --- | --- |
| **Pharmaceutical engineer** |  |
| **Pharmacist** |  |
| **Other** | (Open-text response), hint: Please explain |

**13. Which of the following academic degrees do you hold?**

*(In case more than one, please indicate the last degree you obtained!)*

| **I do not have an academic degree** |  |
| --- | --- |
| **Diploma** |  |
| **Doctorate** |  |
| **License to practice pharmacy** |  |
| **Other** | (Open-text response), hint: Please explain |

**14. Do you hold any additional qualifications related to obtaining drug information?**

*(Multiple answers are possible)*

| **Yes, further specialization training (e.g. specialist pharmacist for drug information).** | (Open-text response),  hint: In case yes, please specify |
| --- | --- |
| **Yes, further education in various areas (e.g. Geriatric pharmacy).** | (Open-text response),  hint: In case yes, please specify |
| **Other** | (Open-text response),  hint: Please explain |
| **No** |  |

**15. How much work experience do you have (in years, after the completion of your training)?**

| **I do not have an academic degree** | up to 1 professional year |
| --- | --- |
| **Diploma** | 2 ­ 5 professional years |
| **Doctorate** | 6 ­ 10 professional years |
| **License to practice pharmacy** | from 11 professional years (and beyond) |

**16. In which pharmaceutical field(s) have you worked within the last 5 years?**

(Multiple answers are possible)

| **1) Community pharmacy** |  |
| --- | --- |
| **2) Hospital pharmacy** |  |
| **3) Research and/or teaching** |  |
| **4) Pharmaceutical industry** |  |
| **5) Authorities and public offices** |  |
| **6) Health insurance companies** |  |
| **7) Federal Armed Forces** |  |
| **8) Others** | (Open-text response), hint: Please explain |

In case participants indicated, to have worked in a community pharmacy, the following questions were displayed.

**17. What is or was your position in the pharmacy(ies)?**

| **Pharmacy owner** |  |
| --- | --- |
| **Branch manager** |  |
| **Employee** |  |
| **Other** | (Open-text response), hint: Please explain |

**18. Are you currently involved in customer counselling in (a) community pharmacy(ies)?**

| **Yes** |  |
| --- | --- |
| **No** |  |

**19. Do you know if your colleagues also participated in this survey?**

| **Several colleagues have participated in this survey** | If yes: How many excluding you? |
| --- | --- |
| **I am the only participant** |  |
| **I do not know** |  |

**Supplementary material S2** Cluster analysis

**Tabl. 2** Cluster analysis of the open-text responses.

| **Area of information needs** | **Variables identified based on participants’ open-text responses** | | **Frequency of basic variables** | |
| --- | --- | --- | --- | --- |
| x= number of participants’ responses | Superordinate variables | Basic variables | Absolute number n | % in relation to x |
| a.1) General individual needs (x= 6) | Information about administration and effectiveness | Practical experience | 1 | 16.7 |
|  |  | Mentioning of OTC-product in medical guidelines | 1 | 16.7 |
|  | General information | Information about conflicts of interest in medical guidelines | 1 | 16.7 |
|  |  | Time since market authorization | 1 | 16.7 |
|  |  | Long-term monitoring data | 1 | 16.7 |
|  | Information about study  characteristics | Independence of study results | 1 | 16.7 |
| a.2) Specific individual needs (x= 5) | Information about product application | Practical experience | 2 | 40.0 |
|  |  | Abuse potential | 1 | 20.0 |
|  |  | Misuse potential | 1 | 20.0 |
|  |  | Usage during pregnancy and while breastfeeding | 1 | 20.0 |
|  | General information | Mechanism of action | 1 | 20.0 |
|  | Information about external evidence | Drug benefit based on independent studies | 1 | 20.0 |
| b) Quality needs  (x = 16) | Characteristics of information sources | Independence | 3 | 18.8 |
|  |  | Clarity | 3 | 18.8 |
|  |  | Conciseness | 3 | 18.8 |
|  |  | Practical relevance | 2 | 12.5 |
|  |  | Transparency | 1 | 6.3 |
|  |  | Comprehensibility | 1 | 6.3 |
|  |  | Simple access | 1 | 6.3 |
|  | Information details | Information about study  characteristics | 5 | 31.3 |
|  |  | Expert opinions | 1 | 6.3 |
|  | Display/layout | (visual) Summaries | 3 | 18.8 |
| c) Utilization needs  (x= 56) | Digital/electronic formats | Webinar | 10 | 17.9 |
|  |  | Written/electronic summaries | 6 | 10.7 |
|  |  | Free of charge, easy to use database | 5 | 8.9 |
|  |  | Extension of pharmacy software | 2 | 3.6 |
|  |  | Extension of ABDA-database | 2 | 3.6 |
|  |  | Electronic learning | 2 | 3.6 |
|  |  | Contributions in social media | 2 | 3.6 |
|  |  | Newsletter | 3 | 5.4 |
|  |  | Application (App)/application software | 3 | 5.4 |
|  |  | Video | 1 | 1.8 |
|  | Analogue formats | Print media/((ring-)binder, loose sheet collection) | 3 | 5.4 |
|  |  | Poster/flyer | 2 | 3.6 |
|  |  | Interactive live events | 1 | 1.8 |
|  |  | Monthly/quarterly published information booklet | 1 | 1.8 |
|  | Audio | Podcast | 9 | 16.1 |
|  | Mixed formats (digital/ analogue) | Specialized magazine | 7 | 12.5 |
|  |  | Educational training | 6 | 10.7 |
|  |  | Discussion forums | 3 | 5.4 |
|  |  | Written text | 2 | 3.6 |
| d) Implication needs  (x= 3) | Information search procedure | Finding independent information sources | 1 | 33.3 |
|  |  | Finding independent studies | 1 | 33.3 |
|  |  | Finding information about the EbPharm methodology/concept | 1 | 33.3 |
| e) Access needs (x= 354) | Implementation of information tools for pharmacists | Database | 95 | 26.8 |
|  |  | Independent, regularly up-dated (online-)information tools/information platforms for pharmacists | 24 | 6.8 |
|  |  | Independent (specialized magazine-) articles/specialized media | 13 | 3.7 |
|  |  | Informative websites | 8 | 2.3 |
|  |  | Educational videos | 6 | 1.7 |
|  |  | Information-application (App)/software | 3 | 0.8 |
|  |  | (online-/print) Specialized media | 3 | 0.8 |
|  | Overall conditions | Easier/unified access through cost reduction/special offers for pharmacists | 35 | 9.9 |
|  |  | Better working conditions | 10 | 2.8 |
|  |  | No/less advertisement by the manufacturer | 4 | 1.1 |
|  |  | Better communication between the professional associations/research institutions and pharmacists in practice | 1 | 0.3 |
|  | Optimization of pre-existing information resources | Linking of evidence-based information/databases with the pharmacy software | 48 | 13.6 |
|  |  | Integration of evidence-based information in ABDA-database | 31 | 8.8 |
|  |  | Overview of external evidence-based sources/databases | 6 | 1.7 |
|  |  | Intuitive search engines/-masks | 4 | 1.1 |
|  |  | Translation of foreign literature | 3 | 0.8 |
|  |  | Integration of evidence-based information in manufacturer’s information tools | 3 | 0.8 |
|  |  | Characteristics of information(-sources)/conciseness | 3 | 0.8 |
|  |  | Data extension of summary of product characteristics (drug label) | 1 | 0.3 |
|  | Preferred information source formats | Short information summaries/overview | 44 | 12.4 |
|  |  | Newsletter | 32 | 9.0 |
|  |  | Guidelines for pharmacists | 6 | 1.7 |
|  |  | Visual display of evidence | 4 | 1.1 |
|  |  | Negative lists | 2 | 0.6 |
|  |  | Manufacturer summaries | 2 | 0.6 |
|  |  | Integration of evidence-based information in social media | 1 | 0.3 |
|  |  | Extensive study reports for pharmacists | 1 | 0.3 |
|  | Training | Educational offers | 27 | 7.6 |
|  |  | Integration of basic EbPharm knowledge in university education | 3 | 0.8 |
|  |  | (Digital) Journal club with exchange of practical experience | 1 | 0.3 |
|  | Infrastructure/conditions for implementation of EbPharm | Implementation of institution for critical appraisal | 23 | 6.5 |
|  |  | Fast information retrieval on the internet/accessibility | 5 | 1.4 |
|  |  | Counselling centre for inquiries | 2 | 0.6 |
|  |  | Information about EbPharm basics | 1 | 0.3 |
|  |  | Audits regarding handling external evidence | 1 | 0.3 |
|  | External evidence | Manufacturer independent studies | 9 | 2.5 |
|  |  | Better (data-)transparency/rules about graphical display of study results | 6 | 1.7 |
|  |  | Studies about comparisons between OTC-products | 3 | 0.8 |
|  |  | Legal requirements for the publication of manufacturer information | 2 | 0.6 |
|  |  | Independent/reliable information sources | 3 | 0.8 |

**Supplementary material S3** Correlation analysis

**Table 3** Correlation between the sociodemographic traits professional experience as well as academic degree and familiarity with EbPharm topics.

| **Sociodemographic trait** | **Familiarity with EbPharm topic** | **Correlation coefficient *Spearman’s rho*** | ***p* Value** |
| --- | --- | --- | --- |
| Professional experience | EbPharm 5-step method | ­0.080 | 0.022* |
|  | PICO-scheme | ­0.205 | <0.001* |
|  | Finding external evidence | ­0.194 | <0.001* |
|  | Critical appraisal of clinical studies | ­0.184 | <0.001* |
|  | Interpretation of study results | ­0.141 | <0.001* |
| Academic degree | EbPharm 5-step method | ­0.001 | 0.973 |
|  | PICO-scheme | ­0.024 | 0.493 |
|  | Finding external evidence | ­0.122 | <0.001* |
|  | Critical appraisal of clinical studies | ­0.108 | 0.002* |
|  | Interpretation of study results | ­0.123 | <0.001* |

* Statistically significant. Each statistically significant correlation coefficient represents a small relationship between the sociodemographic traits and the 3-point Likert-scale concerning familiarity with the EbPharm topics.
